# BMC *Pulmonary Medicine* reviewer acknowledgement 2014

**DOI:** 10.1186/s12890-015-0001-7

**Published:** 2015-03-11

**Authors:** Catia Cornacchia

**Affiliations:** BioMed Central, Floor 6, 236 Gray’s Inn Road, London, WC1X 8HB UK

**Carrie Aaron**

USA

**John Abisheganaden**

Singapore

**Simone Accordini**

Italy

**Ritesh Agarwal**

India

**Daniel Ajona**

Spain

**Mohammed Al Ghobain**

Saudi Arabia

**Mazen Al-Alawi**

Ireland

**Pedro Almagro**

Spain

**Jan Peder Amlie**

Norway

**Benoit Arnould**

France

**Michael Arzt**

Germany

**Elie Azoulay**

France

**Helena Backman**

Sweden

**Mona Bafadhel**

UK

**Ulas Bagci**

USA

**Stefano Baglioni**

Italy

**Chunxue Bai**

China

**Emma Baker**

UK

**Ioannis Bakolis**

UK

**Stefan Balabanov**

Switzerland

**Rui Baptista**

Portugal

**Olga Barbarsh**

Russian Federation

**Gabriele Bassi**

Italy

**Eric Bateman**

South Africa

**Jeremy Beitler**

USA

**Kathleen Bennett**

Ireland

**Pascal Berna**

France

**Laurent Bertoletti**

France

**Rajesh Bhagat**

USA

**Urvashi Bhan**

USA

**Surya Bhatt**

USA

**Andrea Bianco**

Italy

**Lucie Blais**

Canada

**Konrad Bloch**

Switzerland

**Jessica Bon**

USA

**Francesco Bonella**

Germany

**Martina Bonifazi**

Italy

**Berislav Bosnjak**

Austria

**Ynuk Bosse**

Canada

**Apostolos Bossios**

Sweden

**Stephen Bourke**

UK

**Afroditi Boutou**

Greece

**Fulvio Braido**

Italy

**Evan Brittain**

USA

**Raquel Britto**

Brazil

**Malcolm Brodlie**

UK

**Denise Brookes**

Australia

**Dina Brooks**

Canada

**Antonio Bugalho**

Portugal

**Pierre-Regis Burgel**

France

**Jennifer Butcher**

USA

**Scotty Butcher**

Canada

**Elif Cadirci**

Turkey

**Carlo Caffarelli**

Italy

**Shaoxi Cai**

China

**Luigino Calzetta**

Italy

**Bin Cao**

China

**Vera Luiza Capelozzi**

Brazil

**Ralph Caraballo**

USA

**Michele Caraglia**

Italy

**Gaetano Caramori**

Italy

**Annalisa Carlucci**

Italy

**Maria Carrabba**

Italy

**Lucia Cazzoletti**

Italy

**Adrian Ceccato**

Argentina

**James Chalmers**

UK

**Mathias Chamaillard**

France

**Helen Chapel**

UK

**Kenneth Chapman**

Canada

**Davide Chiumello**

Italy

**Chang Won Choi**

Korea, South

**Sanjay Chotirmall**

Ireland

**Shah-Hwa Chou**

Taiwan

**Antonio Clavenna**

Italy

**Amelia Clive**

UK

**Paola Cogo**

Italy

**Harold Collard**

USA

**Adam Collison**

Australia

**Richard Conway**

Ireland

**Liam Cormican**

Ireland

**Antonio Corrado**

Italy

**Cristina Costa**

Italy

**Eduardo Costa**

Brazil

**David Coultas**

USA

**Bianca Cox**

Belgium

**Narelle Cox**

Australia

**Bruno Crestani**

France

**Jane Cross**

UK

**Elliot Crouser**

USA

**Roberto Dantas**

Brazil

**Kaid Darwiche**

Germany

**Theodore Dassios**

UK

**Warren Davidson**

Canada

**Wilfried De Backer**

Belgium

**Francesco De Blasio**

Italy

**Anthony De Soyza**

UK

**Esther De Vries**

Netherlands

**Chris Del Mar**

Australia

**Christophe Delclaux**

France

**Yoshiki Demura**

Japan

**Olufemi Desalu**

Nigeria

**Gaetan Deslee**

France

**Harshad Devarbhavi**

India

**Philippe Devillier**

France

**Philip Diaz**

USA

**Simon Doe**

UK

**Arnau Domenech**

Netherlands

**Frank Domino**

USA

**Massimiliano Don**

Italy

**James Donohue**

USA

**Simone Dore**

Italy

**Victor Dourado**

Brazil

**Silvano Dragonieri**

Italy

**Mark Dransfield**

USA

**Gordon Drummond**

UK

**Orianne Dumas**

France

**Steven Duncan**

USA

**Anthony D’Urzo**

Canada

**Michelle Eakin**

USA

**Anna Eis-Huebinger**

Germany

**Mohamed Elloumi**

Tunisia

**John Engelhardt**

UK

**Ralph Epaud**

France

**Berne Eriksson**

Sweden

**Joan Escarrabill**

Spain

**Francisco Estévez**

Spain

**Mark Everard**

Australia

**Shirley Fabris De Souza**

Brazil

**Alen Faiz**

Netherlands

**Francesco Fanfulla**

Italy

**Fatemeh Fattahi**

Netherlands

**Brigitte Fauroux**

France

**Cristiano Fava**

Italy

**Ademola Fawibe**

Nigeria

**Niki Fens**

Netherlands

**De Benedetto Fernando**

Italy

**Miguel Ferrer**

Spain

**James Fink**

USA

**Claudia Flexeder**

Germany

**Gert Folkerts**

Netherlands

**Marilyn Foreman**

USA

**Stephen Fowler**

UK

**Angela Frank**

USA

**Karl-Josef Franke**

Germany

**Oren Fruchter**

Israel

**En-Qing Fu**

China

**Kazuki Furuhashi**

Singapore

**Taiji Furukawa**

Japan

**Jacques Gaillat**

France

**Miguel Gallego**

Spain

**Gilles Garcia**

France

**Vanessa Garcia Larsen**

UK

**Gail Gauvreau**

Canada

**Chris Gentry**

USA

**Johnson George**

Australia

**William Gerthoffer**

USA

**Giovanni Gherardi**

Italy

**Lisa Giovannini-Chami**

France

**John Gjevre**

Canada

**Rainer Gloeckl**

Germany

**Gabriela Godaly**

Sweden

**Irma Godoy**

Brazil

**Tomasz Golczewski**

Poland

**Lutz Goldbeck**

Germany

**Monica Goldklang**

USA

**Matteo Goldoni**

Italy

**Donna Goodridge**

Canada

**Brian Graham**

Canada

**Ronda Greaves**

Australia

**Catherine Greene**

Ireland

**Matthias Griese**

Germany

**Wolfgang Gruber**

Germany

**Jordan Guenette**

Canada

**Ashleigh Guillaumier**

Australia

**Ersin Gunay**

Turkey

**Mingzhou Guo**

China

**Henrik Gutte**

Denmark

**Stig Hagstad**

Sweden

**Huang Haibo**

China

**Peter Hallas**

Denmark

**Sophie Hambleton**

UK

**Megan Hardin**

USA

**Kim Hare**

Australia

**Dominik Hartl**

Germany

**Apostolia Hatziefthimiou**

Greece

**Andreas Hector**

Germany

**Linnea Hedman**

Sweden

**Craig Hersh**

USA

**Genevieve Hery-Arnaud**

France

**Karen Heslop**

UK

**Catherine Hill**

Australia

**Karen Hind**

UK

**Jonathan Hobson**

UK

**Susan Hodgson**

UK

**Anne Holland**

Australia

**Sakae Homma**

Japan

**Arie Hoogendijk**

Netherlands

**Nobuyuki Horita**

Japan

**Wen-Ni Huang**

Taiwan

**Kewu Huang**

China

**Martin Hug**

Germany

**Rodney Hughes**

UK

**Erik Hulzebos**

Netherlands

**Naoki Inagaki**

Japan

**Naohiro Inohara**

USA

**José Luis Izquierdo Alonso**

Spain

**David Jacobs**

USA

**Alan James**

Australia

**An Soo Jang**

Korea, South

**Susan Janson**

USA

**Thomas Janssens**

Belgium

**Berthold Jany**

Germany

**Dennis Jensen**

Canada

**Cheng Ji**

USA

**Wang Jianchun**

China

**Ane Johannessen**

Norway

**Sebastian Johnston**

UK

**Paul Jones**

UK

**J. Joseph**

USA

**Riitta Kaarteenaho**

Finland

**Hannu Kankaanranta**

Finland

**Stefan Karrasch**

Germany

**Nikoleta Kartali**

Greece

**Parastu Kasaie**

USA

**Bernet Kato**

UK

**Leslie Katzel**

USA

**Thomas Keil**

Germany

**Vanessa Kelly**

USA

**Brian Kent**

UK

**H.A.M. Kerstjens**

Netherlands

**Edward Kerwin**

USA

**Rainer Kiefmann**

Germany

**Jae Yeol Kim**

Korea, South

**Victor Kim**

USA

**Paul King**

Australia

**Sylvia Knapp**

Austria

**Lars Knudsen**

Germany

**Andreas Rembert Koczulla**

Germany

**Manolis Kogevinas**

Spain

**Efstratios Koletsis**

Greece

**Jay Kolls**

USA

**Christos Kontogiorgis**

Greece

**Jukka Koskela**

Finland

**Konstantinos Kostikas**

Greece

**Michael Kreuter**

Germany

**Jerry Krishnan**

USA

**Annemarije Kruis**

Netherlands

**Hiroshi Kubo**

Japan

**Tejaswini Kulkarni**

USA

**Rakesh Kumar**

Australia

**Anna Kurdowska**

USA

**Jessica Kynyk**

USA

**Larry Lands**

Canada

**Arnulf Langhammer**

Norway

**Pierantonio Laveneziana**

France

**Federico Lavorini**

Italy

**Lee Lay Tin**

Singapore

**Solène Le Gal**

France

**David Lederer**

USA

**T-C Lee**

Taiwan

**Augustine Lee**

USA

**Anke Lenferink**

Netherlands

**Steffen Leonhardt**

Germany

**Christophe Leroyer**

France

**Janice Leung**

Canada

**James Lewis**

USA

**Yongde Liao**

China

**Yick Hou Lim**

Singapore

**Hsing-Lin Lin**

Taiwan

**Ann Lindberg**

Sweden

**Kathleen Lindell**

USA

**Jianhong Liu**

China

**Zhangsuo Liu**

China

**Adrian Loerbroks**

Germany

**Anders Løkke**

Denmark

**Emmanuel Lorne**

France

**Teck Boon Low**

Singapore

**Renzo Loyaga-Rendon**

USA

**Thomas Luecke**

Germany

**Bo Lundbã*ƒ*Â¤Ck**

Sweden

**Alex Mackay**

UK

**Kenneth Macleod**

UK

**Stephanie Macneill**

UK

**Brendan Madden**

UK

**Jesper Magnusson**

Sweden

**Rohan Maharaj**

Trinidad and Tobago

**Sara Maio**

Italy

**Subramaniam Malarkannan**

USA

**Fabien Maldonado**

USA

**Giovanni Malerba**

Italy

**Manoj Mammen**

USA

**Lyndon Mansfield**

UK

**Emilio Marangio**

Italy

**Nathaniel Marchetti**

USA

**Darcy Marciniuk**

Canada

**Alessandro Marcon**

Italy

**Tatiana Maron-Gutierrez**

Brazil

**Lorraine Martin**

UK

**Carlos Martinez**

USA

**Miguel Angel Martinez-Garcia**

Spain

**Shin Matsuoka**

Japan

**Joerg Mattes**

Australia

**John Matthews**

USA

**Oscar Mayer**

USA

**David Mcallister**

UK

**Amanda Mccullough**

Australia

**Sharon Mcgrath-Morrow**

USA

**Stephen Mckenna**

UK

**Darren Mcloughlin**

Ireland

**Brent Mcparland**

Australia

**Sanjay Mehta**

Canada

**Silke Meiners**

Germany

**Spyros Mentzelopoulos**

Greece

**Elodie Merlot**

France

**Alessandro Mezzani**

Italy

**Massimo Miniati**

Italy

**Marc Miravitlles**

Spain

**Giuseppe Miserocchi**

Italy

**Howard Mitchell**

Australia

**Susanne Modrow**

Germany

**Kelly Moffitt**

UK

**Seyed Javad Moghaddam**

USA

**Firdaus Aa Mohamed Hoesein**

Netherlands

**Geraldine Moloney**

Ireland

**Ute Mons**

Germany

**Paolo Montuschi**

Italy

**Norman Morris**

Australia

**Stephen Mshana**

Tanzania

**Magdalena Muc**

Poland

**Hiroshi Mukae**

Japan

**David Murdoch**

New Zealand

**Nicola Murgia**

Sweden

**Vanessa Murphy**

Australia

**Michelle Murray**

Ireland

**Marjukka Myllarniemi**

Finland

**Ernest Nadal**

USA

**Lutz Naehrlich**

Germany

**Prasad Nagakumar**

UK

**Atsushi Nambu**

Japan

**Steven Nathan**

USA

**J Alberto Neder**

Brazil

**Nicholas Newman**

USA

**Heber Nielsen**

USA

**Vance Nielsen**

USA

**Carlos Nigro**

Argentina

**Akio Niimi**

Japan

**Peter B Noble**

Australia

**Hiroaki Nomori**

Japan

**Cathal O Broin**

Ireland

**Oisin O’Connell**

Ireland

**Denis O’Donnell**

Canada

**Shinichiro Ohshimo**

Japan

**Brian Oliver**

Australia

**Mario Olivieri**

Italy

**Shane O’Neill**

Ireland

**Ellie Oostveen**

Belgium

**David Orenstein**

USA

**Stylianos Orfanos**

Greece

**Adeola Orogade**

Nigeria

**Christian Osadnik**

Australia

**Caroline Owen**

USA

**Panagiotis Paliogiannis**

Italy

**G Iyer Parameswaran**

USA

**Hae-Sim Park**

Korea, South

**Edwin Roger Parra**

Brazil

**Christopher Pascoe**

Canada

**Franco Pasqua**

Italy

**Neeti Pathare**

USA

**Rodolfo Paula Vieira**

Brazil

**Claudio Pedone**

Italy

**Tatjana Pejcic**

Serbia

**Véronique Pepin**

Canada

**Eanes Pereira**

Brazil

**Antonio Pereira-Vega**

Spain

**Diego Peroni**

Italy

**Lionel Perrier**

France

**Irina Petrache**

USA

**Claudio Pignata**

Italy

**Mariona Pinart**

Spain

**Victor Pinto-Plata**

USA

**Ruben Pio**

Spain

**Rein Jan Piso**

Switzerland

**Tainá Pizzignacco**

Brazil

**Venerino Poletti**

Italy

**Janos Porszasz**

USA

**David Price**

UK

**Mariano Provencio**

Spain

**Nirupama Putcha**

USA

**Biyun Qian**

China

**Xiaoqun Qin**

China

**Stuart Quan**

USA

**Philip Quanjer**

Netherlands

**Alexandra L. Quittner**

USA

**Bradley Quon**

Canada

**Danuta Radzioch**

Canada

**S.Vamsee Raju**

USA

**Fanny Rancière**

France

**Monika Raulf-Heimsoth**

Germany

**Nicolas Regamey**

Switzerland

**Kyndaron Reinier**

USA

**Marie-Pierre Revel**

France

**Julian Rex**

Sweden

**Biarta Rhys-Jones**

Australia

**Matija Rijavec**

Slovenia

**Ger Rijkers**

Netherlands

**Jeremy Road**

Canada

**Fatima Rodrigues**

Portugal

**Josanna Rodriguez**

USA

**Juan Carlos Rodriguez**

Spain

**Micaela Romagnoli**

Italy

**Robert Ross**

USA

**Harry Rossiter**

USA

**Rhonda Rosychuk**

USA

**Kjetil Roth**

Norway

**Maureen Rutten-Van Molken**

Netherlands

**Olli Ruuskanen**

Finland

**Dermot Ryan**

UK

**Christopher Ryerson**

Canada

**Didier Saey**

Canada

**Sejal Saglani**

UK

**Amali Samarasinghe**

USA

**Takuya Samukawa**

Japan

**Claudio Maria Sanguinetti**

Italy

**Pierachille Santus**

Italy

**Ian Sayers**

UK

**Paul Scanlon**

USA

**Tjard Schermer**

Netherlands

**Tamara Schikowski**

Switzerland

**Kerry Schnell**

USA

**Clara Schroedl**

USA

**Erica Schultz**

Sweden

**Richard Schulz**

Germany

**Susanne Schulz**

Germany

**Hayley Scott**

Australia

**Ikuo Sekine**

Japan

**Jacobo Sellares**

Spain

**David Serisier**

Australia

**Waheed Shabbir**

Austria

**Mohamed Shamji**

UK

**Nikhil Sharma**

India

**Sk Sharma**

India

**Dominick Shaw**

UK

**Han-Ming Shen**

Singapore

**Kartik Shenoy**

USA

**Guochao Shi**

China

**Minfeng Shu**

USA

**Chin-Chung Shu**

Taiwan

**Nikos Siafakas**

Greece

**Lori Silveira**

USA

**Donald Silverberg**

Israel

**Patricia Silveyra**

USA

**Jodie Simpson**

Australia

**Sally Singh**

UK

**Marcus Sjödin**

Sweden

**Szymon Skoczynski**

Poland

**Chris Smerecnik**

Netherlands

**Elizabeth Soares**

Brazil

**Xavier Soler**

USA

**Paolo Solidoro**

Italy

**Jinwoo Song**

USA

**Ulrich Specks**

USA

**Malcolm Starkey**

Australia

**Brian Stein**

USA

**Sigurd Steinshamn**

Norway

**Iwona Stelmach**

Poland

**Nikolai Stenfors**

Sweden

**Francois Stephan**

France

**Martina Sterclova**

Czech Republic

**Peter Sterk**

Netherlands

**Alastair Stewart**

Australia

**Kerstin Ström**

Sweden

**Hiromu Suzuki**

Japan

**Nicola Sverzellati**

Italy

**Steve Swain**

USA

**Tomasz Szul**

USA

**Toshinori Takada**

Japan

**Nagio Takigawa**

Japan

**Patricia Talamás-Rohana**

Mexico

**Hiroshige Taniguchi**

Japan

**Claudio Tantucci**

Italy

**Lisete Teixeira**

Brazil

**Paula Tejera**

USA

**Rachel Tham**

Australia

**Signe Timm**

Denmark

**Rabindra Tirouvanziam**

USA

**Nevins Todd**

USA

**Yuji Tohda**

Japan

**Sara Tomassetti**

Italy

**Corey Tomczak**

Canada

**Takahiro Tsuburai**

Japan

**Alice Turner**

UK

**Anne Therese Tveter**

Norway

**Jean Tyrrell**

USA

**Argyris Tzouvelekis**

Greece

**John Upham**

Australia

**Don Urquhart**

UK

**Samuel Valenca**

Brazil

**Françoise Van Bambeke**

Belgium

**Bram Van Den Borst**

Netherlands

**Arnoldus J.R. Van Gestel**

Switzerland

**Rebecca Vanders**

Australia

**Ruud Veldhuizen**

Canada

**Mario Venditti**

Italy

**Alain Vergnenègre**

France

**Diego Viasus**

Colombia

**Eugene Haydn Walters**

Australia

**Peter Wark**

Australia

**George Washko**

USA

**Elizabeth Wasilevich**

USA

**Tomohiro Watanabe**

Japan

**Steven Weinstein**

USA

**James Michael Wells**

USA

**Anja Werno**

New Zealand

**Geertjan Wesseling**

Netherlands

**Jiri Widimsky**

Czech Republic

**Ursula Winzer-Serhan**

USA

**Lisa Wood**

Australia

**Heinrich Worth**

Germany

**Tsuneo Yamashiro**

Japan

**Ian Yang**

Australia

**Ming Yang**

Australia

**Jun Yokota**

Spain

**Robert P Young**

New Zealand

**Susan Yount**

USA

**Zafar Zafari**

Canada

**Warren Zapol**

USA

**Paul Zarogoulidis**

Greece

**Qingxiang Zeng**

Australia

**Jie Zhang**

China

**Lin Zhang**

USA

**Graeme Zosky**

Australia

**Biljana Zvezdin**

Serbia

